# Transient Receptor Potential Channel Polymorphisms Are Associated with the Somatosensory Function in Neuropathic Pain Patients

**DOI:** 10.1371/journal.pone.0017387

**Published:** 2011-03-29

**Authors:** Andreas Binder, Denisa May, Ralf Baron, Christoph Maier, Thomas R. Tölle, Rolf-Detlef Treede, Achim Berthele, Frank Faltraco, Herta Flor, Janne Gierthmühlen, Sierk Haenisch, Volker Huge, Walter Magerl, Christian Maihöfner, Helmut Richter, Roman Rolke, Andrea Scherens, Nurcan Üçeyler, Mike Ufer, Gunnar Wasner, Jihong Zhu, Ingolf Cascorbi

**Affiliations:** 1 Division of Neurological Pain Research and Therapy, Department of Neurology, University Hospital Schleswig-Holstein, Campus Kiel, Kiel, Germany; 2 Institute of Experimental and Clinical Pharmacology, University Hospital Schleswig-Holstein, Campus Kiel, Kiel, Germany; 3 Department of Pain Management, BG Kliniken Bergmannsheil, Ruhr University Bochum, Bochum, Germany; 4 Department of Neurology, Technische Universität München, Klinikum rechts der Isar, München, Germany; 5 Center for Biomedicine and Medical Technology Mannheim, Ruprecht-Karls-University Heidelberg, Mannheim, Germany; 6 Institute of Clinical and Cognitive Neuroscience, Central Institute for Mental Health, Ruprecht-Karls-University Heidelberg, Mannheim, Germany; 7 Department of Anaesthesiology, Ludwig-Maximilians-University München, München, Germany; 8 Department of Neurology, University of Erlangen, Erlangen, Germany; 9 Department of Neurology, University Medical Center, Johannes-Gutenberg-University of Mainz, Mainz and Department of Palliative Care, University of Bonn, Bonn, Germany; 10 Department of Neurology, University of Würzburg, Würzburg, Germany; Istituto Dermopatico dell'Immacolata, Italy

## Abstract

Transient receptor potential channels are important mediators of thermal and mechanical stimuli and play an important role in neuropathic pain. The contribution of hereditary variants in the genes of transient receptor potential channels to neuropathic pain is unknown. We investigated the frequency of transient receptor potential ankyrin 1, transient receptor potential melastin 8 and transient receptor potential vanilloid 1 single nucleotide polymorphisms and their impact on somatosensory abnormalities in neuropathic pain patients. Within the German Research Network on Neuropathic Pain (Deutscher Forscbungsverbund Neuropathischer Schmerz) 371 neuropathic pain patients were phenotypically characterized using standardized quantitative sensory testing. Pyrosequencing was employed to determine a total of eleven single nucleotide polymorphisms in transient receptor potential channel genes of the neuropathic pain patients and a cohort of 253 German healthy volunteers. Associations of quantitative sensory testing parameters and single nucleotide polymorphisms between and within groups and subgroups, based on sensory phenotypes, were analyzed. Single nucleotide polymorphisms frequencies did not differ between both the cohorts. However, in neuropathic pain patients transient receptor potential ankyrin 1 710G>A (rs920829, E179K) was associated with the presence of paradoxical heat sensation (p = 0.03), and transient receptor potential vanilloid 1 1911A>G (rs8065080, I585V) with cold hypoalgesia (p = 0.0035). Two main subgroups characterized by preserved (1) and impaired (2) sensory function were identified. In subgroup 1 transient receptor potential vanilloid 1 1911A>G led to significantly less heat hyperalgesia, pinprick hyperalgesia and mechanical hypaesthesia (p = 0.006, p = 0.005 and p<0.001) and transient receptor potential vanilloid 1 1103C>G (rs222747, M315I) to cold hypaesthesia (p = 0.002), but there was absence of associations in subgroup 2. In this study we found no evidence that genetic variants of transient receptor potential channels are involved in the expression of neuropathic pain, but transient receptor potential channel polymorphisms contributed significantly to the somatosensory abnormalities of neuropathic pain patients.

## Introduction

Neuropathic pain arises after lesions or diseases of the somatosensory nervous system and involves multiple somatosensory phenomena. In addition to spontaneous pain, these include negative and positive symptoms and signs such as hypaesthesia, hypoalgesia, thermal and mechanical hyperalgesia and allodynia [1]. These somatosensory abnormalities and their combination, i.e. the somatosensory profile, may mirror the underlying pain-generating mechanisms [1]. The German Research Network on Neuropathic Pain (DFNS) developed a standardized quantitative sensory testing (QST) battery for somatosensory phenotyping of neuropathic pain patients, including healthy control reference data, facilitating the routine clinical assessment [2], [3].

The understanding of the molecular basis of neuropathic pain has been expanded by the cloning and characterization of the transient receptor potential (TRP) family of voltage-gated ion channels. Among these the TRP vanilloid 1 (TRPV1), TRP melastin 8 (TRPM8) and TRP ankyrin 1 (TRPA1) subfamilies have been found to play important roles in transduction and sensitisation in primary afferent somatosensory neurons [4]. TRPV1 is involved in the transduction of noxious heat and a receptor for capsaicin, thus mediating heat hyperalgesia after stimulation with this hot chilli constituent [5]. It is expressed on small diameter nociceptive neurons, likely to be C-fibers [6]. TRPM8 is also expressed on small and medium diameter sensory neurons, likely to be C and A-delta fibers. It is strongly activated by its agonist menthol and serves as a major cold and cooling transduction channel [7], [8]. TRPA1, activated by its agonists cinnamon and mustard oil, transduces cold and mechanical stimuli. Moreover, on the peptide level it is 20% homologous to TRPV1 and is co-expressed with TRPV1 in a subpopulation of unmyelinated nociceptive neurons suggesting an important role in nociception [9].

Beside these physiological functions, TRPs are thought to play an important role in the generation of neuropathic pain, as demonstrated by in vitro and in vivo animal studies and neurophysiological and psychophysical studies in human experimental pain models and pain patients [10]. TRP channels have been shown to be differentially expressed following experimental nerve injury [11], [12]. Moreover, enhanced expression of TRP channels on neighbouring undamaged fibres following peripheral nerve injury was previously described to regulate pain behaviour [11], [12]. TRPV1 was shown to be involved in the activation and sensitisation of primary sensory neurons to heat, acid and different endogenous inflammatory mediators, leading to heat and mechanical hyperalgesia and spontaneous pain. Likewise TRPM8 contributes to cold pain, cold hyperalgesia and allodynia and TRPA1 leads to activation, sensitisation and facilitation of nociception like TRPV1 [11], [12].

However, the contribution of a genetic variability in TRP channels on pain perception and pain generation in neuropathic pain patients is unknown. In this study we describe the results of an association analysis of polymorphisms in TRPV1, TRPM8 and TRPA1 genes with somatosensory signs of neuropathic pain patients. The aims were (1) to determine the frequencies of TRP variants in a large group of neuropathic pain patients of different aetiology and a cohort of healthy volunteers and (2) to determine the relationship of TRP gene variants and somatosensory function in neuropathic pain patients. Within this study we could demonstrate significant associations between the somatosensory function and TRP gene variants in neuropathic pain patients but no differences in the TRP frequencies compared with healthy volunteers.

## Methods

### Ethics statement

All subjects gave their written informed consent and the study was approved by the ethics committee of the Medical Faculty of the Christian-Albrechts-University Kiel and subsequently the Ethic Committees of the other participating DFNS centers and performed according to the Declaration of Helsinki.

### Study populations

A total of 371 patients with chronic neuropathic pain syndromes, all Caucasians, who participated in the German Research Network on Neuropathic Pain (DFNS; www.neuro.med.tu-muenchen.de/dfns/) were included in the study (210 female, 161 male). The patients suffered from complex regional pain syndrome, postherpetic neuralgia, peripheral nerve injury, trigeminal neuropathy, polyneuropathy, central pain and neuropathies that did not fulfill diagnostic criteria of the above mentioned syndromes as diagnosed by expert physicians. Patient characteristics are listed in Table 1. For comparison of allelic frequencies of target polymorphisms, 253 healthy German volunteers (173 female, mean age 26.8±6.9 years; 80 male, mean age 28.4±7.0 years), all Caucasian, were included.

**Table 1:** Demographic data of patients.

	All neuropathic pain patients	Cluster 1	Cluster 2	p-valuesCluster 1 vs. Cluster 2
N	371	121	120	
Age (years)	56.4±14.4	52.2±13.7	56.6±13.8	p<0.02
Females (%)	57.7	56.1	49.1	p = 0.34
Disease duration (months)	43.9±71.9	39.1±78.6	35.1±47.4	p = 0.63
Pain intensity (0–10 Likert scale NRS)	6.0±2.0	6.0±2.0	6.0±2.0	p = 1.00
Polyneuropathy (n, %)	101 (27.2)	24 (19.8)	41 (35.0)	p<0.02
Post herpetic neuralgia (n, %)	35 (9.4)	7 (5.8)	14 (11.6)	p = 0.16
Painful nerve injury (n, %)	34 (9.2)	13 (10.7)	14 (11.6)	p = 0.98
CRPS (n, %)	87 (23.5)	40 (33.1)	29 (24.1)	p = 0.17
Trigeminal pain (n, %)	57 (15.4)	28 (23.1)	7 (5.8)	p<0.0001
Central pain (n, %)	24 (6.5)	4 (3.3)	7 (5.8)	p = 0.53
Other neuropathy (n, %)	33 (8.9)	5 (4.2)	8 (6.1)	p = 0.56

Values are given as means ± single standard deviation, percentages or absolute values respectively. Pain intensity of the last four weeks was assessed using the numeric rating scale (NRS; 0 = no pain, 10 = worst pain imaginable).

### Phenotypical characterization (quantitative sensory testing)

All patients were phenotypically characterized using standardized quantitative sensory testing (QST) established by the DFNS to determinate somatosensory function and signs of pain [2]. This QST battery consists of seven tests that assess 13 parameters. Briefly, thermal detection thresholds for the perception of cold (CDT) and warm (WDT), thermal pain thresholds for cold (CPT) and heat (HPT), paradoxical heat sensation (PHS), mechanical detection thresholds for touch (MDT) and vibration (VDT), mechanical pain sensitivity including thresholds for pinprick (MPT) and blunt pressure (PPT), a stimulus-response-function for pinprick sensitivity (MPS) and dynamic mechanical allodynia (DMA) as well as pain summation to repetitive pinprick stimuli (wind-up ratio; WUR) were determined in the area of pain. Patient data were included in the study analysis, if at least results of 12 QST tests were available.

### Genetic analysis

Whole genomic DNA was extracted from venous blood samples obtained from patients and volunteers using the Qiagen Gentra Puregene Blood Kit (Qiagen, Hilden, Germany). The final concentration of gDNA was determined spectrophotometrically. Two SNPs in TRPV1, six in TRPM8 and three in TRPA1 genes were identified by literature review in the context of neuropathic pain and selected for detailed association analysis (Table 2). Except of TRPM8 787A>T and TRPM8 790G>C (both synonymous polymorphisms detected in parallel in our genotyping assay), all SNPs were carefully selected based on the assumption that missense variants may have functional consequences (detailed information are included in the Discussion section). Genotyping of the TRP polymorphisms was performed by pyrosequencing according to protocols provided by the manufacturer on a PSQ HS96 platform (Biotage AB, Uppsala, Sweden). Primer sequences and conditions are available on request.

**Table 2:** Significant associations of TRPV1 variants and somatosensory function.

		QST test			
		CDT	HPT	MDT	MPS
**1103C>G**	CC/CG/GG	0.0023^1^			
	CC/CG+GG	0.0019^2^			
	CC+CG/GG	0.0018^2^			
**1911A>G**	AA/AG/GG		0.0059^1^	0.0004^1^	n.s.^1^
	AA/AG+GG		n.s.^2^	<0.0001^2^	0.005^2^
	AA+AG/GG		0.0006^2^	n.s.^2^	n.s.^2^

Significant associations of selected TRPV1 variants with three parameters of quantitative sensory testing (CDT, cold detection threshold, HPT, heat pain threshold, MDT, mechanical detection threshold) in 121 patients belonging to cluster 1. Z-score means of each QST parameter were correlated with genotypes applying a dominant or recessive model.

1 ANOVA test,

2 t-test.

### Statistical analysis

Before statistical analysis the QST values that did not show normal distribution (all except CPT, HPT, VDT and PHS) were transformed logarithmically [2]. Subsequently, Z-scores were calculated, except for DMA and PHS that are given with original values (DMA: 0–100 numeric rating scale; PHS: numbers of PHS, 0–3) [2]. Z-scores above “0” indicate a gain of function (hyperalgesia, allodynia), while a Z-score below “0” indicates a loss of function (hypaesthesia, hypoalgesia). Pathological values were determined according normative data (95% confidence interval = Z-score of 0±1.96). Additionally, DMA and PHS were encoded as dichotomized values (1 = pathological, 0 = within normal range).

In order to identify major subpopulations of patients who are characterized by a typical combination of signs, a cluster analysis was performed in those patients only from whom complete QST results were available (241 of 371 patients, Table 1). The commonly recommended WARD-approach with an Euclidian distance measure was used as described elsewhere [13]. Only those clusters determined within the first WARD fusion algorithm calculation were used. The clusters represent significantly different somatosensory QST Z-profiles demonstrating cluster specific sensory profiles.

The statistical software SPSS 17.0 was employed for all statistical evaluations. Genotype, respectively allele distributions, in the population of patients and healthy controls as well as differences of dichotomised QST values related to target polymorphisms were assessed with Chi-square and Yates corrected Chi-square test respectively. Differences in measured QST parameters (means of Z-scores) and for bi-allelic polymorphisms each subtest and genotype were calculated using analysis of variance (ANOVA). For dominant and recessive models the t-test was used, if applicable. To correct for multiple testing each test was performed using 100,000 permutations.

## Results

### Genotype distribution

The frequency distribution of all polymorphisms investigated was in Hardy-Weinberg equilibrium. Moreover, after correcting for multiple comparisons, the frequency of all SNPs did not differ significantly between neuropathic pain patients, healthy controls and data from the HapMap project (Table S1).

### Association of TRP gene variants to QST Z-scores

**Figure 1:**
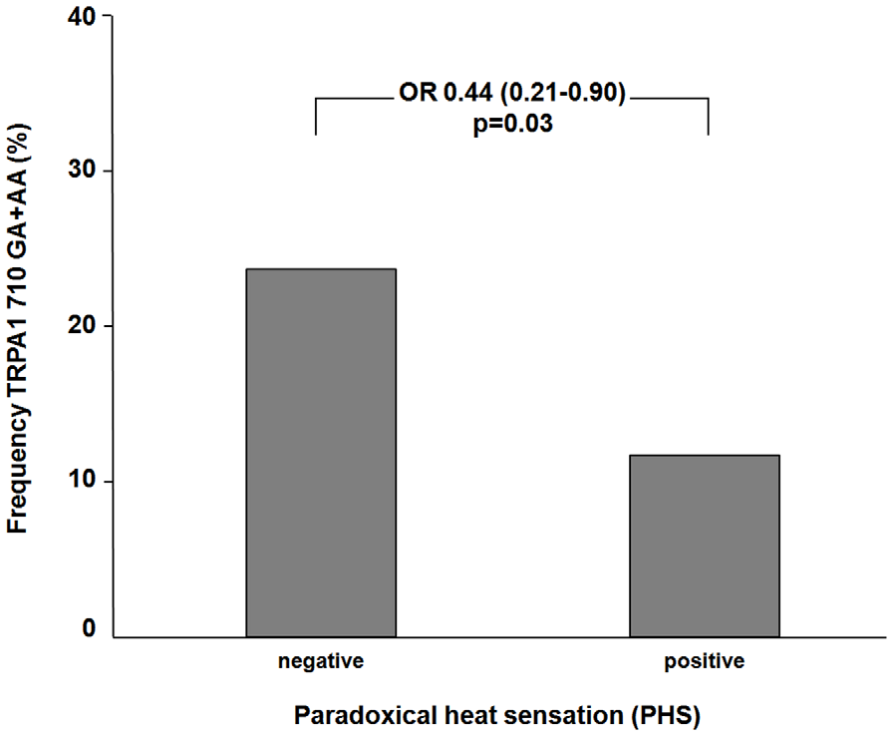
Association of TRPA1 710G>A to appearance of paradoxical heat sensation. Patients with positive paradoxical heat (PHS) showed a lower frequency of variant TRPA1 710 genotypes (GA+AA carriers) compared to patients with negative PHS (odds ratio 0.44, 95% confidence interval 0.21–0.90, p = 0.03).

First, within the group of patients who suffered from paradoxical heat sensation (PHS) heterozygous and homozygous carriers of the TRPA1 710G>A variant were significantly under-represented as compared to neuropathic pain patients without PHS (odds ratio 0.44, 95% confidence interval 0.21–0.90, p = 0.03) (Fig. 1). Second, homozygous variant carriers (GG) of the TRPV1 1911A>G polymorphism exhibited significant cold hypoalgesia compared with heterozygous or wild type carriers (CPT Z-scores: −0.13±1.10, 0.19±1.20, and 0.18±1.14, respectively; p = 0.003).

### Somatosensory profiles of subpopulations

**Figure 2:**
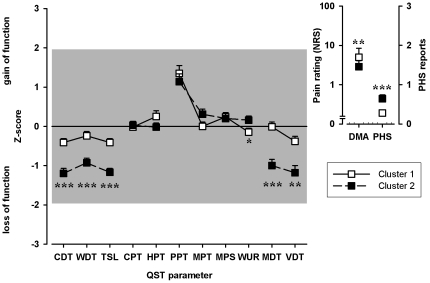
Somatosensory profiles of cluster 1 and cluster 2. Cluster 1 is characterized by a predominant preserved somatosensory function whereas cluster 2 exhibits relative loss of small fiber and large fiber function (lower Z-scores for CDT, WDT, TSL, MDT, VDT and WUR respectively). Values are given as arithmetic mean and standard error of mean. The grey marked area displays the normative range (Z-scores between +/−1.96). P-values: *<0.05, **<0.01, ***<0.001.

Hierarchical cluster analysis detected two main clusters (Fig. 2). Cluster 1 includes a group of patients with mainly preserved sensory function, while cluster 2 was characterized by a relative loss of small and large fiber function as detected by significant lower Z-scores of CDT, WDT, TSL, MDT (p<0.0001), VDT (p = 0.002) and a higher Z-score of WUR (p = 0.027) in patients who belong to cluster 2 as compared to cluster 1. DMA and PHS differed significantly between the two clusters. Pathological PHS occurred significantly more frequent in cluster 2 (odds ratio 3.44, 95% CI 1.78–6.68, p<0.0001) and DMA was more frequent in cluster 1 (odds ratio 2.30, 95% CI 1.30–4.07, p = 0.004).

### Association of TRP gene variants to QST Z-scores of subpopulations

Z-scores of all QST tests were stratified to all TRP variants investigated in the entire sample and in both clusters. After correcting for multiple comparisons, two TRPV1 polymorphisms, 1911A>G and 1103C>G, were identified that had a significant relationship with the somatosensory function in cluster 1 (Table 2).

**Figure 3:**
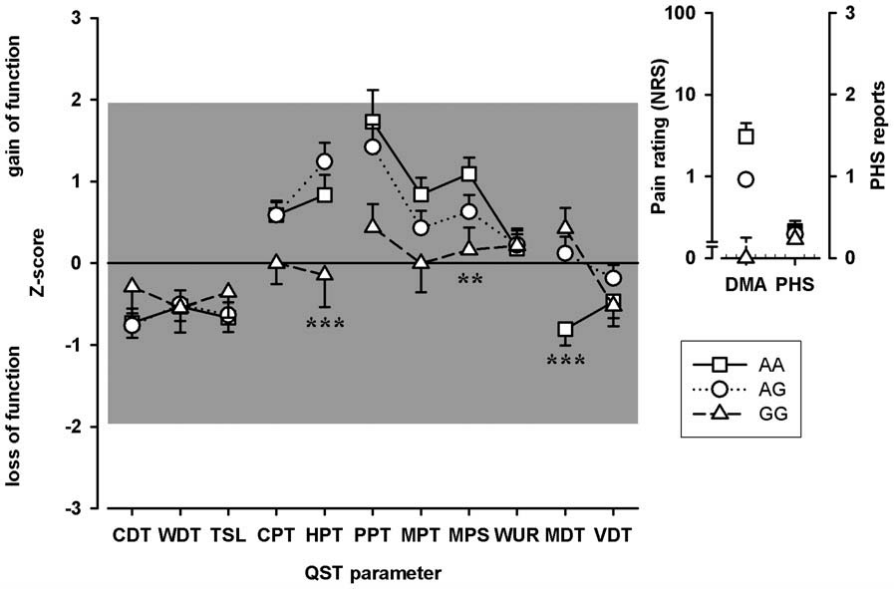
The influence of TRPV1 1911 A>G on QST parameters in cluster 1. The influence of the TRPV1 1911A>G genotype on the somatosensory profile as detected by QST in the patients of cluster 1 is shown. Homozygote variant carriers expressed significant lower, i.e. normal, Z-scores for HPT (p = 0.0006). The second genotype-affected QST parameter is MDT. The higher Z-scores for MDT, i.e. less mechanical hypaesthesia to non-painful mechanical stimuli, correlated with the presence of G-variant allele in TRPV1 1911A>G (p<0.0001). Third, lower Z-scores of mechanical pain sensitivity (MPS), i.e. less pinprick hyperalgesia, were associated with the presence of at least one G-variant allele in TRPV1 1911A>G (p = 0.005). Values are given as arithmetic mean and standard error of mean. P-values are shown for significant genotype-phenotype associations: **<0.01, ***<0.001.

**Figure 4:**
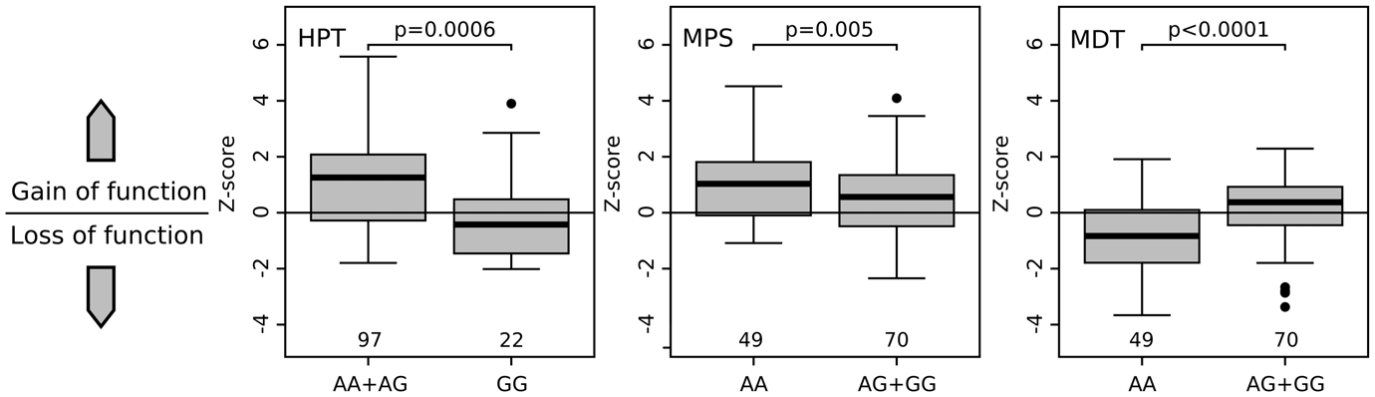
Associations of TRPV1 1911 A>G with HPT, MPS and MDT in cluster 1. Z-scores of heat pain threshold (HPT), mechanical pain sensitivity (MPS) and mechanical detection threshold (MDT) stratified to TRPV1 1911G>A. **HPT:** Homozygous variant GG carriers exhibited significantly lower, i.e. normal, Z-scores than carriers of the heterozygote or wild type genotype, which tended to show heat hyperalgesia. **MPS:** Presence of at least one G-variant allele resulted in a decreased Z-score indicating less pinprick hyperalgesia. **MDT:** Presence of at least one G-variant allele was associated with less mechanical hypaesthesia to non-painful mechanical stimuli resulting in higher Z-scores. Z-scores are given as median with quartiles and ranges, p-values were calculated with t-test and corrected for multiple testing by permutation testing.

The TRPV1 1911A>G polymorphism was significantly associated with altered heat pain thresholds (HPT). Neuropathic pain patients being heterozygote or with wild type TRPV1 1911G genotype (AA or AG) tended to show heat hyperalgesia whereas TRPV1 1911G homozygotes exhibited significantly higher, i.e. normal heat pain thresholds (p = 0.0006; Fig. 3, 4). TRPV1 1911A>G was also identified to be associated significantly to mechanical pain sensitivity (MPS; p = 0.005). The presence of at least one G-variant allele was associated with lower, i.e. normalized mechanical pain sensitivity to pinprick stimuli (MPS; Fig. 3, 4). Moreover, TRPV1 1911A>G wild type carriers (AA) showed higher mechanical detection thresholds (MDT), i.e. mechanical hypaesthesia, than variant subjects (p<0.0001; Fig. 3, 4).

**Figure 5:**
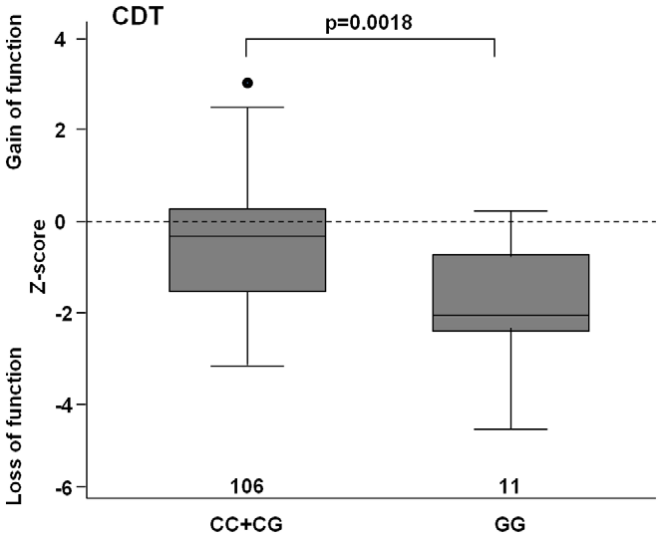
The effect of TRPV1 1103 C>G on detection of non-painful cold. The box plots show the cluster 1 specific association of TRPV1 1103C>G SNP with the cold detection threshold (CDT). Homozygous variant carriers exhibited cold hypaesthesia, i.e. lower Z-score, compared with heterozygous or wild type carriers. Z-scores are given as median with quartiles and range. P-values were calculated with t-test and corrected for multiple testing by permutation testing.

In contrast to TRPV1 1911A>G, the 1103C>G SNP was significantly associated with cold detection threshold (CDT, p = 0.0023). Homozygous variant carriers (GG) exhibited cold hypaesthesia compared with heterozygous or wild type carriers (p = 0.0018; Fig. 5).

**Figure 6:**
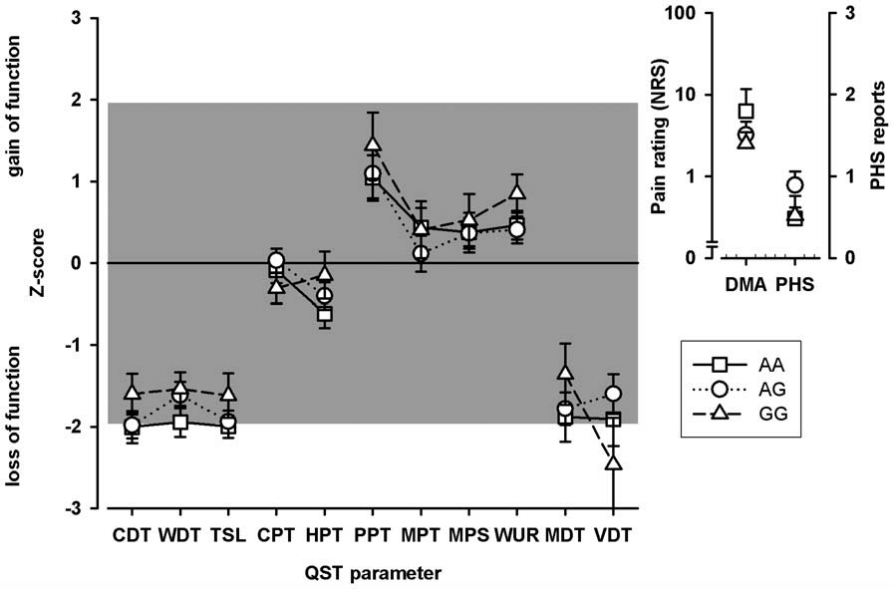
The influence of TRPV1 1911 A>G on QST parameters in cluster 2. The influence of the TRPV1 1911A>G genotype on the somatosensory profile as detected by QST in the patients of cluster 2 is shown. The hereditary impact on somatosensory functions was detectable in cluster 1 only. No significant associations were detected in cluster 2. Values are given as arithmetic mean and standard error of mean.

Analyses within cluster 2 did not show any genotype-dependent association with any QST parameter (Fig. 6). Due to the small sample size, no analysis of possible associations of the different neuropathic pain syndromes and QST parameters were performed.

## Discussion

The major finding of this study is that TRP channel polymorphisms modulate the somatosensory function in neuropathic pain patients. Since there were no significant differences in the allelic distribution between neuropathic pain patients and healthy volunteers it appears unlikely that the variants play a role as susceptibility factors of chronic neuropathic pain.

### Association of TRPA1 710G>A with paradoxical heat sensation

Different mechanisms contribute to paradoxical heat sensation, i.e. the perception of painful heat when a cold stimulus is applied. First, A-delta fiber impairment, clinically manifest in a disturbed cold sensation, is thought to disinhibit C-nociceptors by mechanisms of gate control in the dorsal horn of the spinal cord leading to PHS [14], [15]. This hypothesis is supported by observations in the experimental human pain model of selective A-fiber blockade and in patients with peripheral neuropathy [14], [16]. Second, dysfunction of thalamic cells leads to disinhibition of central heat transmission pathways, resulting in PHS e.g. in patients with multiple sclerosis [17], [18]. The frequency of TRPA1 710G>A variant carriers was higher in PHS cases. Although the functional significance of this polymorphism has not yet been investigated in vitro so far, it can be hypothesized that the amino acid exchange from hydrophilic glutamate to hydrophobic lysine in codon 237 may result in a conformational change of the protein secondary structure. With respect to the clinical observation, a loss of function appears to be likely and thus result in an impairment of cold nociceptor sensitivity. This might lead to a decreased input to the central nociceptive pathway that is the same pathway for the conduction of heat and PHS. This would, according to the hypotheses described above, lead in turn to a lower likelihood of an effective disinhibition and thereby PHS.

Similar to the TRPV1 receptor, TRPA1 is known to sensitize sensory neurons after nerve injury and contribute to altered pain sensation in a familial episodic pain syndrome [19]. Therefore, wild type carriers of the active TRPA1 710 might have a higher propensity for sensitisation of nociceptors peripherally and/or centrally, increasing the likelihood of PHS compared to carries of the variant genotype. Interestingly, a psychophysical study in CRPS patients demonstrated that PHS is a frequent finding in acute CRPS [20] and inflammation is thought to be a dominant cause of sensitisation in acute CRPS patients [21]. Possibly TRPA1 contributes to this proposed mechanism in CRPS, since TRPA1 is activated in models of inflammatory pain and leads to pain and hyperalgesia [11], [12].

### Association of TRPV1 1911A>G with cold pain threshold

At first sight the association of TRPV1 variants to CPT may sound paradox, since TRPV1 is known as a heat sensor. However, our observation is in line with earlier findings by Kim and coworkers [22] who demonstrated in healthy volunteers that homozygote TRPV1 1911A>G variant carriers exhibited longer cold withdrawal times to ice water immersion of one hand, i.e. higher cold pain tolerance. In our study, the Z-score of the cold pain threshold in 1911GG carriers was lower than in AG and AA carriers (−0.13±1.10 vs. 0.19±1.20 and 0.18±1.14 respectively) indicating cold hypoalgesia.

TRPV1 1911A>G causes an isoleucine-valine exchange, both hydrophobic amino acids. Though the variant 1911G was associated with elevated protein levels in a human kidney cell line expression system [23], the investigation of functional responses to capsaicin, pH and temperature in whole-cell patch-clamp and calcium imaging experiments suggests no critical alteration of receptor structure and function with respect to this receptor variant [24]. Another explanation for the effect of TRPV1 on cold pain thresholds could be a possible interaction of TRPV1 and the cold sensor TRPA1 since both are co-expressed in sensory neurons [11], [12]. However, functional and linkage studies are currently lacking. Therefore our finding requires further confirmation.

### Associations in phenotypic subpopulations

We were able to allocate the phenotypically characterized patients to two different clusters. Patients in a cluster share sensory symptoms and signs and thus may share similar underlying pathological mechanisms [25]. Interestingly, in a study that investigated nerve and skin preparations of patients who suffered from various neuropathic pain syndromes, TRP channels have been found to be differentially expressed [26], thus supporting the approach of stratification. One may hypothesize that in those patients in whom TRPs are still preserved or increasingly expressed SNP polymorphisms contribute to the somatosensory function, whereas those who show a depletion of e.g. nerve growth factor (NGF), the membrane density of TRP channels is likely to be decreased [27]. Interestingly, Facer and coworkers [26] found TRPV1 to be reduced in diabetic neuropathy patients. Accordingly, in cluster 2 where the number of polyneuropathy patients was significantly higher as in cluster 1, we did not find any association between TRP variants and somatosensory function.

As mentioned above, TRPV1 1911A>G has not been shown to alter TRPV1 function in vitro or in vivo [24]. However, in this study it clearly modified different somatosensory functions in patients allocated to cluster 1.

TRPV1 is a heat sensor and contributes to the sensitisation of peripheral nociceptors in neuropathic pain [11], [12]. The corresponding clinical sign is heat hyperalgesia [1]. Peripheral sensitisation of TRPV1 sensitive nociceptors in turn leads to sensitisation of the central nociceptive system and clinically to mechanical hyperalgesia [1]. Due to central adaptive mechanisms tactile hypaesthesia accompanies mechanical hyperalgesia [28]. Thus, each sensory finding is compatible with neurobiological and neuropharmacological mechanisms that can also be studied in human surrogate models of neuropathic pain, e.g. capsaicin model that involve a reversible modulation of the nociceptive system [29], [30]. These observations are in line with our findings on the TRPV1 1911 genotype. Wild types or heterozygotes showed heat hyperalgesia, mechanical hyperalgesia and mechanical hypaesthesia in comparison to variant carriers that showed normalised sensitivity to heat pain (HPT), mechanical pain detection (MPT) and tactile sensation (MDT) (Figure 3, 4). Thus, the variant TRPV1 1911A>G may prevent from peripheral and secondary central sensitisation in patients with chronic neuropathic pain.

The second TRPV1 SNP of interest, namely TRPV1 1103C>G was significantly associated with cold hypaesthesia. The SNP causes a methione-isoleucine exchange and in-vitro investigation demonstrated an increased channel activity [23]. However since no effect of TRPV 1103C>G on HPT was observed, the question arises if this observation might be explained by a functional interaction of TRPM8 and TRPV1 that leads to increased functional inhibition of the TRPM8 by this SNP [31], [32].

### Study limitations

There are three main limiting factors of this study. First, the number of SNP selected for this study was limited to missense variants. No intronic, promoter, 5′- or 3′-UTR variants were included as no association to pain or functional characterization could be shown so far [33]. The functional significance of the missense variants found to be associated with sensory function in our study, has not yet been characterized in vitro. Thus the findings are only based on a genetic association analysis. However, our statistical analysis was conducted conservatively using - among others - permutation corrections that still led to significant associations and thus makes chance findings less likely. Second, these associations need to be cross-validated in larger samples. However, to our knowledge, there is currently no patient cohort available outside the DFNS that used a comparable phenotyping method. Hopefully, within the European Pain Consortium, a second cohort will evolve, making soon further genetic association studies and cross-validation possible. Third, no conclusions on the role of these TRP polymorphisms in other ethnic groups can be drawn, as it was recently demonstrated that African Americans exhibit differential response to the topical application of the TRPV1 agonist in capsaicin compared to three other ethic groups [34].

### Research and clinical perspectives

Our results demonstrate the need for further research on the functionality of TRP genetic variants on sensory function and nociception. Further on, our results open new avenues for the treatment of neuropathic pain patients. First, specific TRPV1, TRPM8 and TRPA1 antagonists are under research as new therapeutic options, with the analgesic TRPV1 agonist capsaicin already available [35]–[37]. Second, it has to be clarified how far genetic variants of TRP channels may account for response, non-response or symptom-specific efficacy. This would suggest the subgrouping of patients along clinically relevant markers when they enter clinical trials and follow-up trials [38]. Moreover, variants identified here may serve as detectable risk factors for the course of the disease and gene tests on specific SNPs might serve as predictors of pain qualities in the future.

## Supporting Information

Table S1 Frequencies of selected TRP channel gene variants in 371 neuropathic pain patients and 253 healthy volunteers. P-values between genotypes of patients and controls are corrected for multiple testing using 100,000 permutations.
(DOC)
