# Supplementary material for: Transient Receptor Potential Channel Polymorphisms Are Associated with the Somatosensory Function in Neuropathic Pain Patients
Source: PLoS One. 2011 Mar 29;6(3):e17387. doi: 10.1371/journal.pone.0017387 (PMC3066165; doi:10.1371/journal.pone.0017387)
Supplement: Table S1 — Frequencies of selected TRP channel gene variants in 371 neuropathic pain patients and 253 healthy volunteers. P-values between genotypes of patients and controls are corrected for multiple testing using 100,000 permutations. (DOC) [file pone.0017387.s001.doc]

**Table S1:** **Frequencies of TRP channel gene variants.**

| **Gene** | **mRNA position** | **rs number** | **Amino acid exchange** | **Genotype** | **Frequency (%)** | | | **p-value** |
| --- | --- | --- | --- | --- | --- | --- | --- | --- |
|  |  |  |  |  | **HapMap** | **Patients** | **Controls** |  |
| **TRPA1** | 182C>T | rs13268757 | R3C | CC | 70.0 | 71.1 | 72.0 | n.s. |
|  |  |  |  | CT | 30.0 | 28.7 | 27.6 |  |
|  |  |  |  | TT | 0.0 | 0.2 | 0.4 |  |
|  | 710G>A | rs920829 | E179K | GG | 78.3 | 76.3 | 80.4 | n.s. |
|  |  |  |  | GA | 21.7 | 21.9 | 18.0 |  |
|  |  |  |  | AA | 0.0 | 1.8 | 1.6 |  |
|  | 3228A>G | rs959976 | H1018R | AA | 61.7 | 66.7 | 69.1 | n.s. |
|  |  |  |  | AG | 35.0 | 29.7 | 29.3 |  |
|  |  |  |  | GG | 3.3 | 3.6 | 1.6 |  |
| **TRPV1** | 1103C>G | rs222747 | M315I | CC | 66.7 | 55.9 | 52.4 | n.s. |
|  |  |  |  | CG | 30.0 | 37.0 | 40.7 |  |
|  |  |  |  | GG | 3.3 | 7.1 | 6.9 |  |
|  | 1911A>G | rs8065080 | I585V | AA | 41.7 | 38.8 | 33.2 | n.s. |
|  |  |  |  | AG | 48.3 | 44.8 | 48.0 |  |
|  |  |  |  | GG | 10.0 | 16.4 | 18.8 |  |
| **TRPM8** | 780G>C | rs13004520 | R247T | GG | 88.3 | 88.1 | 92.4 | n.s. |
|  |  |  |  | GC | 11.7 | 11.9 | 6.8 |  |
|  |  |  |  | CC | 0.0 | 0.0 | 0.8 |  |
|  | 787A>T | rs2890163 | P249P | AA | 98.3 | 98.6 | 98.8 | n.s. |
|  |  |  |  | AT | 1.7 | 1.4 | 1.2 |  |
|  |  |  |  | TT | 0.0 | 0.0 | 0.0 |  |
|  | 790G>C | rs11562975 | L250L | GG | 81.7 | 80.1 | 81.2 | n.s. |
|  |  |  |  | GC | 18.3 | 19.9 | 18.4 |  |
|  |  |  |  | CC | 0.0 | 0.0 | 0.4 |  |
|  | 792A>G | rs17868387 | Y251C | AA | 88.3 | 88.3 | 92.0 | n.s. |
|  |  |  |  | GA | 11.7 | 11.7 | 7.2 |  |
|  |  |  |  | GG | 0.0 | 0.0 | 0.8 |  |
|  | 1296G>A | rs7593557 | S419N | GG | 86.7 | 87.2 | 91.1 | n.s. |
|  |  |  |  | GA | 13.3 | 12.4 | 7.6 |  |
|  |  |  |  | AA | 0.0 | 0.4 | 1.3 |  |
|  | 2235C>T | rs17862932 | T732I | CC | 98.3 | 99.6 | 99.6 | n.s. |
|  |  |  |  | CT | 1.7 | 0.4 | 0.4 |  |
|  |  |  |  | TT | 0.0 | 0.0 | 0.0 |  |
